# Laboratory Medicine

**DOI:** 10.1155/2013/269194

**Published:** 2013-05-14

**Authors:** Mina Hur, Andrew St. John, Antonio La Gioia

**Affiliations:** ^1^Department of Laboratory Medicine, Konkuk University School of Medicine, 120-1 Neungdong-ro, Hwayang-dong, Gwangjin-gu, Seoul 143-729, Republic of Korea; ^2^Australian Research Council, Canberra, ACT, Australia; ^3^Italian Society of Clinical Biochemistry and Molecular Biology, Milan, Italy

Clinical laboratories have a vital role to play in translating research findings into clinically useful measurements. This involves assessment of new procedures to ensure that they are analytically valid and also continually integrating the findings from new studies into routine practice. These important tasks are covered in this special issue on laboratory medicine which includes a wide variety of laboratory-related topics as illustrated by three review articles and 12 research papers. The first review article by D.-H. Ko et al. comprehensively examines the methods available for haptoglobin typing and discusses the characteristics, clinical applications, and limitations of each method. A. Noto et al. describe the role of neutrophil gelatinase-associated lipocalin (NGAL) for managing acute kidney injury and the potential benefits derived from the combined clinical use of urine NGAL and metabolomics in kidney disease. The third review by E. Urrechaga et al. looks at new laboratory biomarkers for hypochromia including their clinical significance and utility in daily practice.

Several papers examine issues in diagnostic hematology, one of the traditional areas of laboratory testing. Y. Nam et al. assess hypercoagulability using a relatively new thrombomodulin-induced thrombin generation assay (TGA) in patients with liver cirrhosis. They show that, although routine coagulation tests did not detect the thrombotic tendency in cirrhosis, the TGA could detect hypercoagulability in cirrhosis. The paper by M. Hur et al. emphasizes that prothrombin time international normalized ratio (INR) measurements by point-of-care testing coagulometers still need to be confirmed with INR measurements in the laboratory. H. R. Lee et al. evaluate the relationship between mean platelet volume (MPV) and characteristics of cord blood (CB) units and shows that MPV may be one of the most useful parameters to assist with making decisions about the priority for processing specimens in the cord blood bank. F. Chongliang et al., in their interesting study on complete blood counts, suggest that a model that uses levels of neutrophils, lymphocytes, and platelets is potentially useful in the objective evaluation of survival time or disease severity in unselected critically ill patients.

Three interesting papers come from the areas of serology/immunology, chemistry, and microbiology. K. Lee et al. evaluate the overall efficacy of reverse sequence screening for syphilis (RSSS) and examine the practical issues associated with the routine investigation of syphilis. The study by M. Han et al. analyzes the degree of concordance between the various multiple allergen simultaneous test assays and a reference method. J. Gervasoni et al. evaluate various assay kits for 25-hydroxyvitamin D2/D3 and show that they have acceptable agreement with liquid chromatography-tandem mass spectrometry.

Five papers deal with the growing area of molecular diagnostics. T. Kaewphinit et al. combined a loop-mediated isothermal amplification method with a chromatographic lateral-flow dipstick and show that it can specifically and rapidly detect the IS6110 gene of *M. tuberculosis*. S. Kim et al. introduced a new allele-specific real-time PCR system for *TPMT* genotyping, which can be used to improve the efficacy and safety of thiopurine treatments in clinical practice. R. Januchowsk et al. analyzed *MDR* gene expression in drug-resistant ovarian cancer cell lines. They suggest that it is possible to predict cross-resistance to other drugs when the classical *MDR*, which is correlated with P-gp expression, is involved. S. M. Hwang et al. evaluated the human platelet antigen (HPA) genotype and/or the CD109 mRNA expression in various human cell types. They demonstrate that the 4-1BB signal pathway plays a key role in organ transplantation tolerance. Lastly the paper by Y. Shi et al. demonstrates that gene silencing of 4-1BB by RNA interference inhibits the acute rejection in rats with liver transplantation.

We hope that these papers in this special issue of BioMed Research International will help to confirm the important role that clinical laboratories play in translational research and the need to continuously update laboratory practice as new findings and developments appear.

*Mina Hur* *Andrew St. John* *Antonio La Gioia*

